# About an observation of cerebral abscess encysted

**DOI:** 10.11604/pamj.2017.28.251.12480

**Published:** 2017-11-21

**Authors:** Noukhoum Koné, Sambou Soumaré

**Affiliations:** 1 Service de Neurochirurgie, Centre Hospitalier de Kiffa, Mauritanie; 2 Centre de Radiologie, d’imagerie médicale, Centre Hospitalier de Saint Brieuc, France

**Keywords:** Cerebral abscess, cyst, MRI

## Image in medicine

The cerebral abscess represents 2% of the intracranial lesions of the adult, 17% of the child. 35% of abscesses develop before the age of 15 years. It evolves in several phases (pre-suppurative encephalitis, purulent collection without shell, abscess collected with fine capsule, abscess collected with thick shell). We report the case of a 14-year-old girl with no specific pathological history except a neglected oral infection episode; has been present for 4 weeks with headache, vomiting, tonic-clonic convulsive seizures in a febrile context. On examination there is a proportional right hemiparesis with a motor force rated at 3/5 without facial participation, a fever at 39.5°c. The biological examination revealed a leucocytosis (17 000/mm^3^) predominantly neutrophilic (81%). The brain CT Scan with injection of iodinated contrast medium in axial (A) and coronal (B) sections revealed an isodense occupying process, with a left parietal seat, with annular rehabitation and partitioned, measuring 23.8mm x 20mm and presenting an edema in the satellite. Brain MRI was found 3 weeks later in sequences, T1 (C), T2 Flair (D, E), T1 gado in axial section a volumetric increase of the occupying process with a significant deviation from the line of the median structures. A craniotomy with evacuation of the abscess (F, H) and excision of the cyst wall (G) was performed. The bacteriological examination of the pus was negative. Anatomopathological examination of the cyst wall revealed non-specific inflammatory necrotic tissue. An intravenous antibiotherapy was established in post-operative stages, the clinical course proved to be significantly favorable.

**Figure 1:**
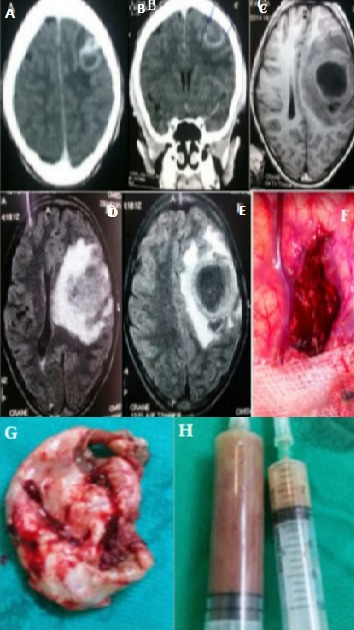
Brain CT Scan with injection of iodinated contrast medium in axial (A) and coronal (B) sections revealed an isodense occupying process, with a left parietal seat, with annular rehabitation and partitioned , measuring 23.8mm x 20mm and presenting an edema in the satellite. The brain MRI at 3 weeks later in sequences, T1 (C), T2 Flair (D, E) in axial section, volumetric increase of the occupant process with a significant deviation from the line of the median structures

